# Alteration of the Premature tRNA Landscape by Gammaherpesvirus Infection

**DOI:** 10.1128/mBio.02664-20

**Published:** 2020-12-15

**Authors:** Jessica M. Tucker, Aaron M. Schaller, Ian Willis, Britt A. Glaunsinger

**Affiliations:** a Department of Plant and Microbial Biology, University of California, Berkeley, California, USA; b Department of Molecular and Cell Biology, University of California, Berkeley, California, USA; c Department of Biochemistry, Albert Einstein College of Medicine, Bronx, New York, USA; d Department of Systems and Computational Biology, Albert Einstein College of Medicine, Bronx, New York, USA; e Howard Hughes Medical Institute, University of California, Berkeley, California, USA; Duke University Medical Center

**Keywords:** RNA polymerase III, gammaherpesvirus, tRNA

## Abstract

Transfer RNAs (tRNAs) are transcribed by RNA polymerase III (RNAPIII) and play a central role in decoding our genome, yet their expression and noncanonical function remain understudied. Many DNA tumor viruses enhance the activity of RNAPIII, yet whether infection alters tRNA expression is largely unknown. Here, we present the first genome-wide analysis of how viral infection alters the tRNAome. Using a tRNA-specific sequencing method (DM-tRNA-seq), we find that the murine gammaherpesvirus MHV68 induces global changes in premature tRNA (pre-tRNA) expression, with 14% of tRNA genes upregulated more than 3-fold, indicating that differential tRNA gene induction is a characteristic of DNA virus infection. Elevated pre-tRNA expression corresponds to increased RNAPIII occupancy for the subset of tRNA genes tested; additionally, posttranscriptional mechanisms contribute to the accumulation of pre-tRNA species. We find increased abundance of tRNA fragments derived from pre-tRNAs upregulated by viral infection, suggesting that noncanonical tRNA cleavage is also affected. Furthermore, pre-tRNA accumulation, but not RNAPIII recruitment, requires gammaherpesvirus-induced degradation of host mRNAs by the virally encoded mRNA endonuclease muSOX. We hypothesize that depletion of pre-tRNA maturation or turnover machinery contributes to robust accumulation of full-length pre-tRNAs in infected cells. Collectively, these findings reveal pervasive changes to tRNA expression during DNA virus infection and highlight the potential of using viruses to explore tRNA biology.

## Abstract (precis)

Viral infection can dramatically change the gene expression landscape of the host cell, yet little is known regarding changes in noncoding gene transcription by RNA polymerase III (RNAPIII). Among these are transfer RNAs (tRNAs), which are fundamental in protein translation, yet whose gene regulatory features remain largely undefined in mammalian cells.

## INTRODUCTION

The elegant design of the tRNA decoder, an adaptor molecule that converts genetic information into protein, was proposed over 60 years ago (1). Although tRNAs were the first noncoding RNAs described, our understanding of tRNA biology, especially their functions outside protein translation and what dictates their expression, remains limited. This is partly due to the difficulties in applying RNA sequencing and analysis platforms to tRNAs, but recent methodological advances have resulted in a surge of new studies that help to resolve these complexities (2–4). Noncanonical functions of tRNA transcripts have been described, namely, the discovery that both premature and mature tRNAs undergo fragmentation into shorter RNAs with numerous regulatory functions in response to stress, cancer, and viral infection (reviewed in references 5 and 6). Given the evidence that the ∼400 predicted tRNA genes in mammalian genomes are differentially expressed across cell lines (7–9) and that viral infection can lead to posttranscriptional modulation of tRNAs (10, 11), defining the tRNAome under various conditions will be central to advancing our understanding of tRNA function, canonical and otherwise.

RNA polymerase III (RNAPIII) transcribes tRNA genes and exhibits enhanced activity during infection with DNA viruses (12–17), many of which carry their own RNAPIII genes. Though RNAPIII activation stimulates expression of viral RNAPIII genes, concomitant accumulation of host RNAPIII transcripts can trigger antiviral immune responses. For example, the induction or misprocessing of the RNAPIII-generated host 5S RNA pseudogene and vault RNA transcripts during herpes simplex virus 1 (HSV-1) and Kaposi's sarcoma-associated herpesvirus (KSHV) infections, respectively, activates RNA sensing pattern recognition receptors (18, 19). The RNAPIII-driven B2 SINE (short interspersed nuclear element) family of retrotransposons are also induced and sensed during infection with the murine gammaherpesvirus MHV68, although MHV68 has uniquely evolved to hijack components of the antiviral NF-κB signaling pathway to instead promote viral gene expression and replication (20–24). While it is clear that induced RNAPIII transcripts can have functional consequences during infection, little is known about the mechanisms underlying the selectivity of RNAPIII activation, including identification of the specific RNAPIII genes induced by viral infection on a genome-wide scale.

DNA viruses upregulate RNAPIII transcription by increasing the abundance of limiting RNAPIII transcription factor complexes specific for certain RNAPIII promoter types (reviewed in reference 25). RNAPIII genes have one of three different promoters that recruit a unique combination of the transcription factor complexes TFIIIA (Gtf3a), TFIIIB (TBP [TATA-binding protein], Brf1 or Brf2, and Bdp1), and TFIIIC (Gtf3c1 to -6) or SNAPc (Snapc1 to -5) plus RNAPIII for gene expression (26). tRNAs use the abundant RNAPIII promoter type II, which consists of internal A and B boxes that bind TFIIIC and ultimately recruit TFIIIB and RNAPIII to initiate transcription. 7SL, short interspersed nuclear elements (SINEs), vault RNAs, and viral RNAPIII genes also use type II promoters. 5S rRNA genes have an internal type I promoter, which binds TFIIIA (Gtf3a) and requires TFIIIC, TFIIIB, and RNAPIII for transcription. In contrast, type III promoters found in U6 and 7SK genes have external promoters that are TFIIIC-independent and bind the SNAPc complex and a unique TFIIIB complex containing Brf2 protein, rather than Brf1. Defining virally induced RNAPIII transcript classes and/or transcription factor complexes has been essential for understanding how viruses enhance RNAPIII activity.

The breadth of tRNA function in cells and the fact that many DNA viruses increase RNAPIII activity suggest that regulation of tRNA expression may be a significant point of control during infection. The repertoire of differentially expressed tRNA genes during DNA virus infection is not known, although individual tRNA transcripts are upregulated during Epstein-Barr virus (EBV) and simian virus 40 (SV40) infection (12, 27). Here, we present the first genome-wide analysis of how infection alters tRNA abundance by using the model gammaherpesvirus MHV68. Using a tRNA-specific sequencing method (DM-tRNA-seq) and analysis pipeline, we found that lytic MHV68 infection leads to a ≥3-fold increase in premature tRNA (pre-tRNA) levels for approximately 14% of tRNA genes. Notably, infection-induced pre-tRNAs do not appear to undergo canonical maturation, as mature tRNA levels remain largely constant, although upregulated pre-tRNAs can be further cleaved into shorter tRNA fragments (tRFs). The accumulation of pre-tRNAs occurs through a combination of transcriptional induction, as evidenced by increased RNAPIII occupancy at activated loci, and posttranscriptional mechanisms. In particular, pre-tRNA accumulation is linked to the activity of the viral endonuclease muSOX/ORF37, which cleaves host mRNAs and downregulates protein synthesis during infection in a process called host shutoff. Given that muSOX is not required for RNAPIII recruitment to tRNA genes, we instead hypothesize that degradation of host mRNAs depletes factors involved in tRNA processing and turnover, contributing to the accumulation of pre-tRNAs. The alterations in tRNA expression during MHV68 infection described here highlight the potential of using viruses to explore mechanistic details of tRNA maturation and turnover.

## RESULTS

### Pre-tRNAs increase in abundance in MHV68-infected cells.

**FIG 1:**
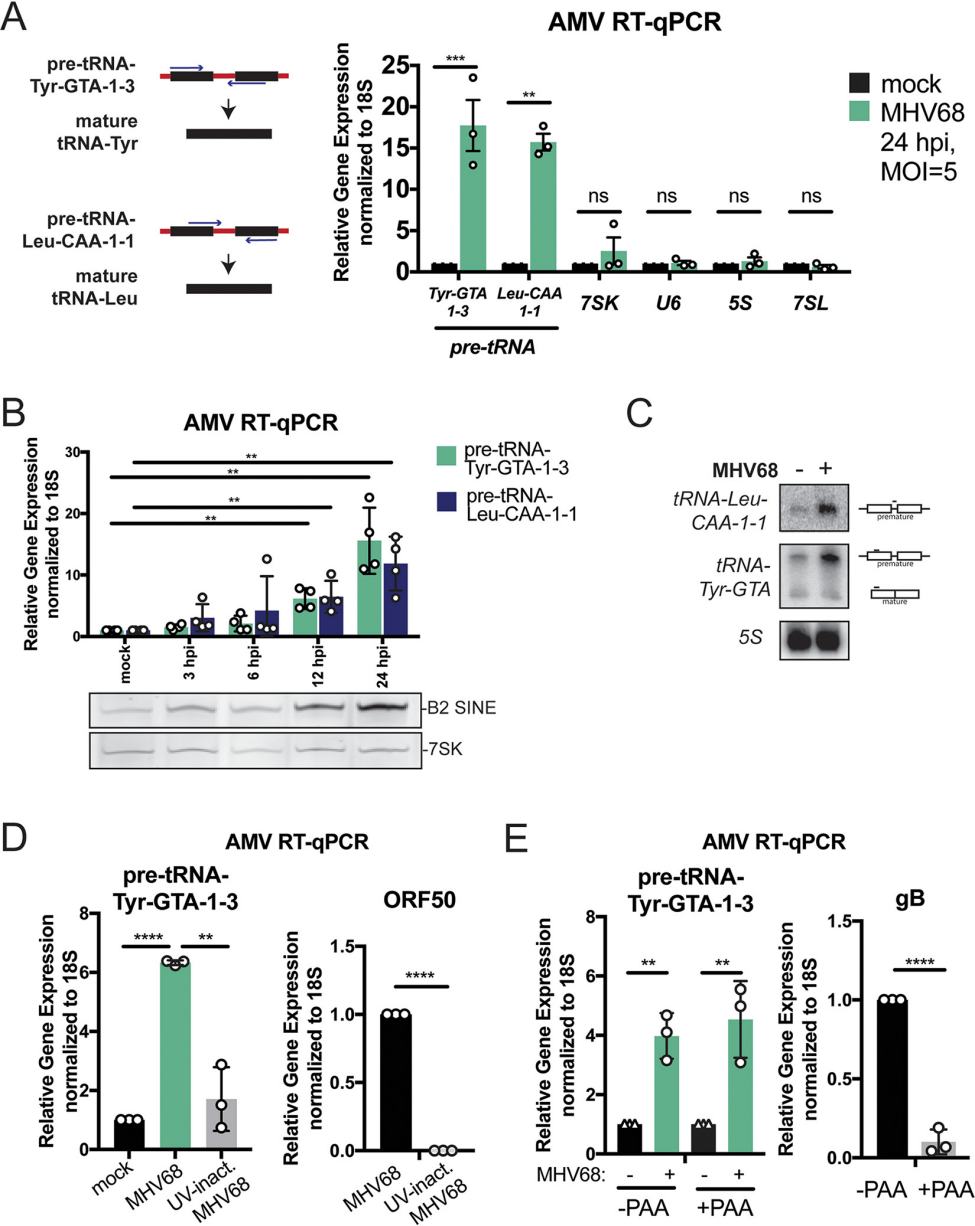
MHV68 infection results in increased pre-tRNA expression. (A) Pre-tRNA-Tyr-GTA-1-3 and pre-tRNA-Tyr-Leu-CAA-1-1 are transcribed with flanking and intron sequences (depicted in red), which are removed during maturation. Pre-tRNAs were detected with forward or reverse primers with 3′ ends complementary to the intron by avian myeloblastosis virus (AMV) RT-qPCR. Relative abundance of pre-tRNAs and other RNAPIII transcripts was measured by RT-qPCR in mock-infected and MHV68-infected NIH 3T3 cells. Expression was normalized to 18S rRNA and compared to values from mock-infected cells. (B) RNA was extracted from NIH 3T3 cells following a time course of MHV68 infection and used for both AMV RT-qPCR to detect pre-tRNAs and primer extension to detect B2 SINE and 7SK transcripts. (C) Northern blotting was performed on RNA from mock or MHV68-infected MC57Gs. Transcripts produced from tRNA-Leu-CAA-1-1 were detected with intron- and exon-specific probes. tRNA-Tyr transcripts were detected with a probe that anneals with a conserved 5′ region in the tRNA-Tyr family. Probe binding is depicted on the right. (D) RNA from mock- and MHV68-infected MC57G cells ± UV inactivation was used for AMV RT-qPCR. ORF50 is an early viral gene. (E) Mock- and MHV68-infected MC57G cells were treated with 200 ng/μl phosphonoacetic acid (PAA) to block viral DNA replication, and extracted RNA was used for RT-qPCR analysis. *gB* is a late viral gene whose expression is dependent on successful viral DNA replication. All infections were done at an MOI of 5 for 24 h unless otherwise noted. RT-qPCR experiments were done in triplicate at a minimum. Error bars show the standard deviation (SD), and statistics were calculated using an unpaired *t* test on raw Δ*C_T_* values. ns, not significant. **, *P* < 0.01; ***, *P* < 0.001; ****, *P* < 0.0001.

Given that MHV68 infection upregulates RNAPIII-driven B2 SINEs (20), which are evolutionarily related to tRNAs, we hypothesized that gammaherpesviral infection may also alter cellular tRNA expression. We first measured pre-tRNA levels by quantitative reverse transcriptase PCR (RT-qPCR), using primer sets that were specific for premature tRNAs (pre-tRNAs) produced from intron-containing tRNA-Tyr and tRNA-Leu genes (Fig. 1A). During MHV68 infection of NIH 3T3 mouse fibroblasts, pre-tRNA levels increased by greater than 15-fold (Fig. 1A). This induction appeared specific to tRNAs, as other RNAPIII transcripts (*7SK*, *U6*, *5S*, and *7SL*) did not show differential expression during infection. This is in contrast to Epstein-Barr virus, a gammaherpesvirus that is associated with increased expression of many RNAPIII gene classes due to upregulation of TFIIIB/TFIIIC transcription factor complex subunits at both the mRNA and protein levels (12). Accordingly, we did not detect changes in TFIIIB/C subunit mRNA levels upon MHV68 infection (see Fig. S1 in the supplemental material), and in fact these transcripts were less abundant during infection. This is in line with the general downregulation of host mRNA transcripts that occurs during MHV68-induced host shutoff (28). A time course of MHV68 infection showed that pre-tRNA-Tyr and pre-tRNA-Leu levels were increased by 12 h postinfection (hpi), with their levels accumulating through late stages of infection at 24 hpi (Fig. 1B). Notably, the timing of pre-tRNA induction corresponded well with the induction pattern for B2 SINEs. Finally, we also confirmed that pre-tRNA-Tyr and pre-tRNA-Leu are upregulated by Northern blotting, a method that does not rely on cDNA production (Fig. 1C). Mature tRNA-Tyr transcript levels remained constant during infection, suggesting that infection-induced pre-tRNAs may not be processed into mature tRNAs.

10.1128/mBio.02664-20.1 FIG S1 The levels of mRNAs encoding TFIIIB and TFIIIC subunits are decreased upon MHV68 infection. RT-qPCR was performed on MC57G cells infected at an MOI of 5 for 24 h and were done in triplicate. Error bars show SD, and statistics were calculated using an unpaired *t* test on raw Δ*C_T_* values. ns, not significant. *, *P* < 0.05. Download FIG S1, TIF file, 1.3 MB. Copyright © 2020 Tucker et al. 2020 Tucker et al. This content is distributed under the terms of the Creative Commons Attribution 4.0 International license.

Given the kinetics of induction, we hypothesized that events occurring mid to late infection were responsible for pre-tRNA accumulation, as opposed to a cellular response to viral binding and entry. In agreement with this hypothesis, infection of MC57G fibroblasts with UV-inactivated MHV68 did not lead to pre-tRNA-Tyr induction (Fig. 1D). Additionally, wild-type (WT) MHV68 infection of the MC57G fibroblast cell line led to an induction similar to that in NIH 3T3 cells. UV inactivation prevented postentry viral gene expression, as demonstrated by the absence of expression of the immediate early viral gene ORF50. To narrow down the gene class responsible for the kinetics of tRNA induction, we performed infections in the presence of phosphonoacetic acid (PAA), which blocks viral DNA replication and prevents the expression of viral late genes, such as *gB*. Although late gene expression was inhibited, pre-tRNA-Tyr accumulated to similar levels in cells infected in the presence of PAA (Fig. 1E). Thus, one or more effects of early viral gene expression underlie pre-tRNA accumulation during MHV68 infection. Together, these data demonstrate that early viral gene expression drives the increased abundance of host pre-tRNAs.

### DM-tRNA-seq reveals upregulation of premature tRNAs during infection.

Extensive base modification of tRNAs can prevent reverse transcription of full-length cDNA products. Although there are some modifications present in pre-tRNAs, they likely have not undergone extensive modification and therefore are more efficiently amplified relative to mature tRNAs during reverse transcription (4, 29). Amplification of full-length cDNA products from tRNAs can be improved by using the highly processive thermostable group II intron containing reverse transcriptase (TGIRT) (30) and by partial removal of tRNA strong-stop modifications by treatment with AlkB demethylases (2, 3). To define the extent of tRNA upregulation genome-wide, we generated sequencing libraries using TGIRT- and AlkB-treated small RNA (a method called DM-tRNA-seq [3]) in four biological replicates of mock-infected versus MHV68-infected MC57G cells. Our analysis pipeline was designed to distinguish reads from pre-tRNAs and mature tRNAs, as our preliminary data with tRNA-Leu and tRNA-Tyr suggested that MHV68 may elevate pre-tRNA but not mature tRNA levels (Fig. 1C). Reads were first aligned to mature tRNA sequences, which contained predicted tRNA genes from tRNAscan-SE (31, 32) modified by intron removal and the addition of mature -CCA 3′ ends, yielding counts for mature tRNAs. The remaining reads were aligned to a masked genome appended with pre-tRNA sequences, including intact introns and 50 nucleotides (nt) of upstream and downstream flanking sequence. Thus, classification as a pre-tRNA required the absence of a 3′-CCA and the presence of 5′ leader, intron, or 3′ trailer sequence.

tRNAscan-SE yields a list of genes containing both true tRNAs and tRNA-like sequences, such as pseudo-tRNAs and B2 SINEs. Among these, we focused on the high-confidence set of tRNA genes as defined by the Genomic tRNA Database (GtRNAdb) (*n* = 405) (31, 32). Due to unique genomic sequences found in 5′ leader and 3′ trailers, pre-tRNA reads were less likely to multimap to the genome and were used as a proxy for expression of the respective tRNA genes. Of the 405 predicted high-confidence tRNA genes, we were able to map pre-tRNAs originating from 313 tRNA genes by using SALMON (Data Set S1) (33). Additionally, we used a different mapping strategy analyzing only the uniquely mapping reads in order to more confidently identify the locus of origin for expressed pre-tRNAs, although this strategy may underestimate the total number of expressed pre-tRNAs. Restricting the data to uniquely mapping reads yielded a total of 244 tRNA genes with detectable pre-tRNAs from infected cells.

10.1128/mBio.02664-20.6 DATA SET S1 Differential gene expression analysis output from DM-tRNA-seq comparing MHV68-infected versus mock-infected MC57G fibroblasts. Download Data Set S1, XLSX file, 0.01 MB. Copyright © 2020 Tucker et al. 2020 Tucker et al. This content is distributed under the terms of the Creative Commons Attribution 4.0 International license.

**FIG 2:**
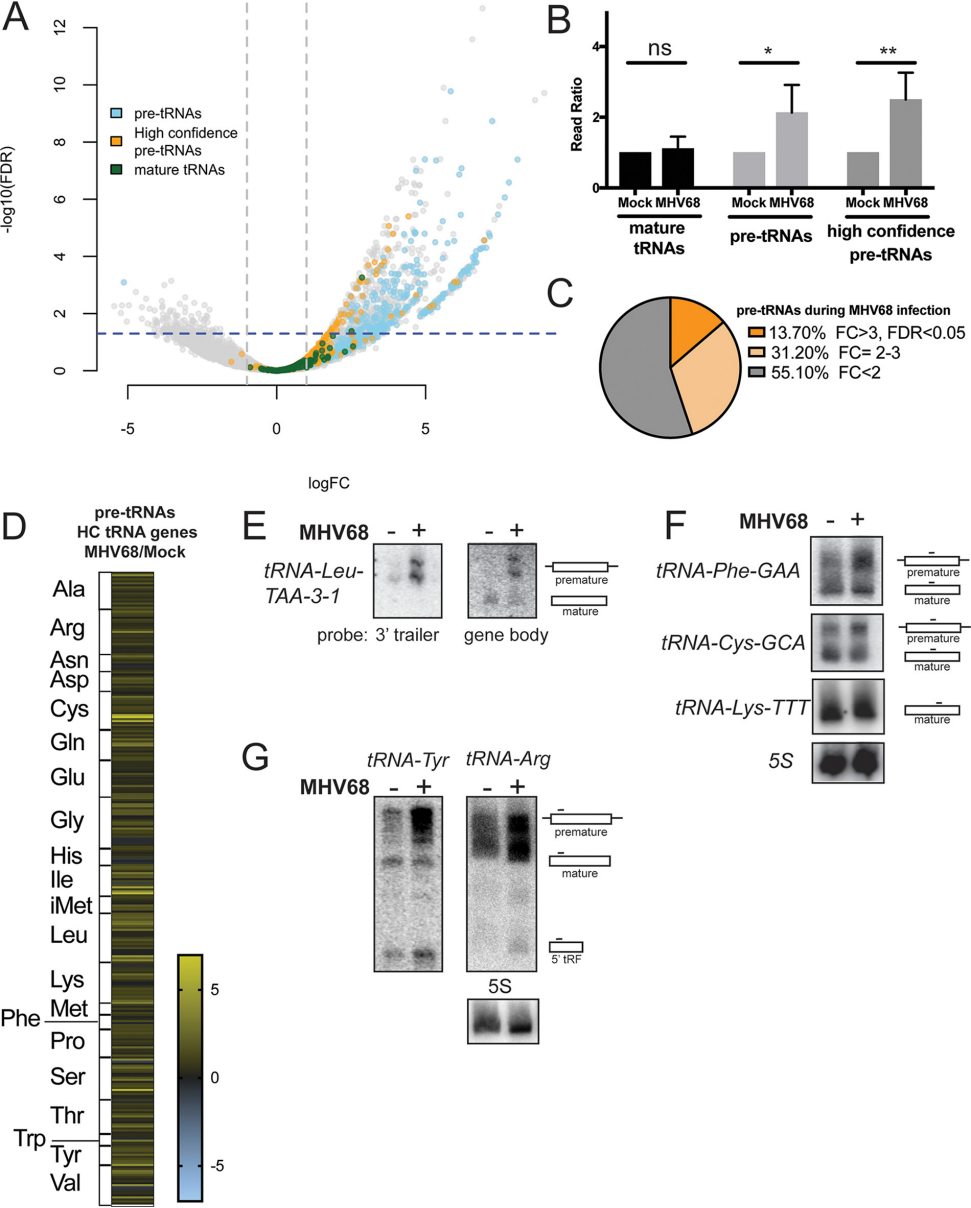
DM-tRNA-seq reveals differential expression of the pre-tRNAome upon infection. (A) Differential gene expression analysis from DM-tRNA-seq was plotted as fold change (FC) versus the false-discovery rate (FDR). Pre-tRNAs (high confidence in yellow, others in blue), mature tRNAs (high confidence only in red), and other small RNA reads (gray) are annotated, and the dotted lines represent the boundaries of FC = 2 and FDR = 0.05. High confidence refers to the tRNA gene set predicted by GtRNAdb. (B) Raw read counts for mature tRNAs, pre-tRNAs, or high-confidence pre-tRNAs from DM-tRNA-seq of MHV68-infected MC57Gs were compared to those of uninfected cells. Error bars show SD, and statistics were calculated using an unpaired *t* test on raw read counts from four replicates. (C) A pie chart shows the percentage of high-confidence pre-tRNAs detected by DM-tRNA-seq that were significantly upregulated (dark orange, FC > 3 and FDR < 0.05) versus upregulated but not significant (light orange, FC > 2 and FDR > 0.05) versus unregulated (gray, FC < 2). (D) A heat map of log_2_ FCs of pre-tRNA levels by DM-tRNA-seq by isotype. (E) RNA from mock-infected or MHV68-infected MC57G cells was used for Northern blotting with probes specific for either the 3′ trailer or gene body of tRNA-Leu-TAA-3-1. (F) RNA from mock- or MHV68-infected MC57G cells was used for Northern blotting with probes specific for the gene body of the tRNA-Phe-GAA, tRNA-Cys-GCA, and tRNA-Lys-TTT families. Probes detected tRNAs from multiple loci. (G) RNA from mock- or MHV68-infected MC57G cells was used for Northern blotting with probes specific for the 5′ exon of the tRNA-Tyr-GTA and tRNA-Arg-TCT families. Probes detected tRNAs from multiple loci. All infections were done in MC57G fibroblasts at an MOI of 5 for 24 h. ns, not significant. *, *P* < 0.05; **, *P* < 0.01.

Differential gene expression analysis revealed that pre-tRNAs are more abundant during MHV68 infection (Fig. 2A, blue and orange circles). In fact, raw read count ratios alone (sum of total reads per replicate compared to mock infection) displayed an increase in pre-tRNA reads present in infected cells compared to uninfected cells (Fig. 2B). It is likely that many of the highly expressed pre-tRNA genes (Fig. 2A, blue circles) that are not in the high-confidence tRNA gene list (Fig. 2A, orange circles) are SINE retrotransposons, as B2 SINEs are robustly induced during infection (20, 34). Focusing on high-confidence tRNA genes, we found many of these loci produce more pre-tRNAs during infection (Fig. 2A, orange circles, and Fig. 2C). Yet, while total levels of pre-tRNAs were higher in infected cells, we observed selectivity in which tRNA genes were upregulated. Of the high-confidence pre-tRNAs detectably expressed in uninfected MC57G cells, approximately one-fifth were significantly upregulated during MHV68 infection, depending on the mapping strategy used. Figure 2C shows results with SALMON: 43 out of 313 (13.7%) pre-tRNAs were upregulated with a false-discovery rate (FDR) of <0.05. Mapping unique reads with Bowtie2 yielded 63 out of 244 (25.8%) upregulated pre-tRNAs with an FDR of <0.05 (Data Set S1). However, a general trend of increased expression of pre-tRNAs was observed, as judged by a fold change (FC) greater than 2 by both mapping methods: for SALMON, 141 out of 313 (45%) pre-tRNAs (Fig. 2C; Data Set S1); for unique mappers with Bowtie2, 158 out of 244 (64.8%) pre-tRNAs (Data Set S1). In general, at least one member of each tRNA isotype (tRNAs encoding the same amino acid) were present in the differential hits, but not all isodecoders (tRNAs with the same anticodon) were represented (Fig. 2D; see Fig. S2 in the supplemental material). A triple-A-box motif that is present in each RNAPIII-driven TMER (tRNA-miRNA-encoded RNA) locus carried by MHV68 and in 10% of host tRNA genes (35) was not enriched in the differential hits (see Fig. S3 in the supplemental material).

10.1128/mBio.02664-20.2 FIG S2 A variety of tRNA isotypes are upregulated at the pre-tRNA level in response to MHV68 infection. Pre-tRNAs detected by DM-tRNA-seq from each tRNA isodecoder are shown in black, while those with increased pre-tRNA expression in response to MHV68 are shown in pink. Most isotypes (tRNAs encoding the same amino acid), but not all isodecoders (tRNAs with same anticodon), are represented. Download FIG S2, TIF file, 1.6 MB. Copyright © 2020 Tucker et al. 2020 Tucker et al. This content is distributed under the terms of the Creative Commons Attribution 4.0 International license.

10.1128/mBio.02664-20.3 FIG S3 A triple-A-box motif is not enriched in MHV68-induced tRNA genes. The triple-A-box motif (TRGYNNARNTGGTRGARNAGNNG) identified in MHV68 TMERs is present in ∼11% of mouse high-confidence tRNA genes, as previously reported (35, 69). Both uniquely mapped and SALMON-identified MHV68-induced tRNA genes (*n* = 79 genes) were analyzed for the triple-A-box motif. Download FIG S3, TIF file, 1.3 MB. Copyright © 2020 Tucker et al. 2020 Tucker et al. This content is distributed under the terms of the Creative Commons Attribution 4.0 International license.

Unlike pre-tRNAs, the levels of most mature tRNAs did not change in a statistically significant manner, suggesting that the upregulated pre-tRNAs do not undergo canonical maturation (see Data Set S1 in the supplemental material). In fact, only two high-confidence tRNA genes (tRNA-Lys-TTT-2-2 and tRNA-Leu-TAA-3-1) showed significant differential expression at the level of the mature tRNA transcript (Fig. 2A, green circles, FDR of <0.05). To validate these findings, we designed Northern blot probes to the 3′ trailer or gene body specific to transcripts originating from tRNA-Leu-TAA-3-1 (Fig. 2E). While pre-tRNA transcripts were elevated from the tRNA-Leu-TAA-3-1 gene in infected cells, levels of mature tRNAs did not change upon infection. We were not able to design a probe specific for tRNA-Lys-TTT-2-2 transcripts, but using a probe that recognizes members of the tRNA-Lys-TTT family did not show any differences in mature tRNA-Lys-TTT expression during MHV68 infection (Fig. 2F). Similarly, other tRNA families that we tested by Northern blotting, including the tRNA-Phe-GAA and tRNA-Cys-GCA families, showed upregulation only at the level of the full-length pre-tRNA (Fig. 2F).

### MHV68 induces differential expression of pre-tRNA fragments.

Studies have demonstrated that differential expression of tRNAs, either when comparing different mammalian cell types or upon deletion of the RNAPIII negative regulator Maf1, is not associated with major changes in steady-state mature tRNA levels (36, 37). Instead, increased tRNA expression largely seems to affect pre-tRNA and/or tRNA fragment (tRF) levels. DM-tRNA-seq analysis revealed increased expression of pre-tRNAs, but the size selection we used for this pipeline (50 to 200 nt) did not allow for interrogation of tRF expression. To investigate whether MHV68 infection leads to changes in tRF levels, we performed Northern blot analysis using probes that detect Tyr or Arg tRFs derived from the 5′ end of the pre-tRNA transcripts, previously reported in mouse embryonic fibroblasts (38) (Fig. 2G). We found that Tyr and Arg tRF abundance was modestly increased upon infection with MHV68.

The reported activities for tRFs are strikingly diverse, rendering functional predictions difficult, particularly under conditions such as infection, where a multitude of different fragments may be produced (11, 39). Nonetheless, we explored possible activity for the Tyr tRF, as its overexpression has been previously reported to enhance p53 activation through phosphorylation at serine 18 (S18) in response to oxidative stress (38). Because MHV68 infection also leads to p53 activation through phosphorylation at S18 (40), we examined whether this phenotype was linked to pre-tRNA induction. However, depletion of the TFIIIB subunit Brf1 required for polymerase III (Pol III) activity did not alter the p53 phosphorylation status in MHV68-infected cells (see Fig. S4 in the supplemental material). Thus, while MHV68-induced pre-tRNAs may undergo processing into small RNA fragments, whether these harbor specific functions (for example, in specific cell contexts) remains unknown. Regardless of their function, virus-induced pre-tRNA accumulation provides a platform for dissecting the mechanisms underlying tRNA gene expression control.

10.1128/mBio.02664-20.4 FIG S4 p53 phosphorylation at S18 during MHV68 infection is not dependent on Brf1 expression. NIH 3T3 mouse fibroblasts were nucleofected twice with either control or Brf1-targeting siRNAs and then infected with MHV68 at an MOI of 5 for 24 h. Total protein was blotted for Brf1, p53, p53-S18, and GAPDH. Download FIG S4, TIF file, 2.1 MB. Copyright © 2020 Tucker et al. 2020 Tucker et al. This content is distributed under the terms of the Creative Commons Attribution 4.0 International license.

### Increased levels of RNA polymerase III are found at tRNA genes associated with increased pre-tRNA expression during infection.

**FIG 3:**
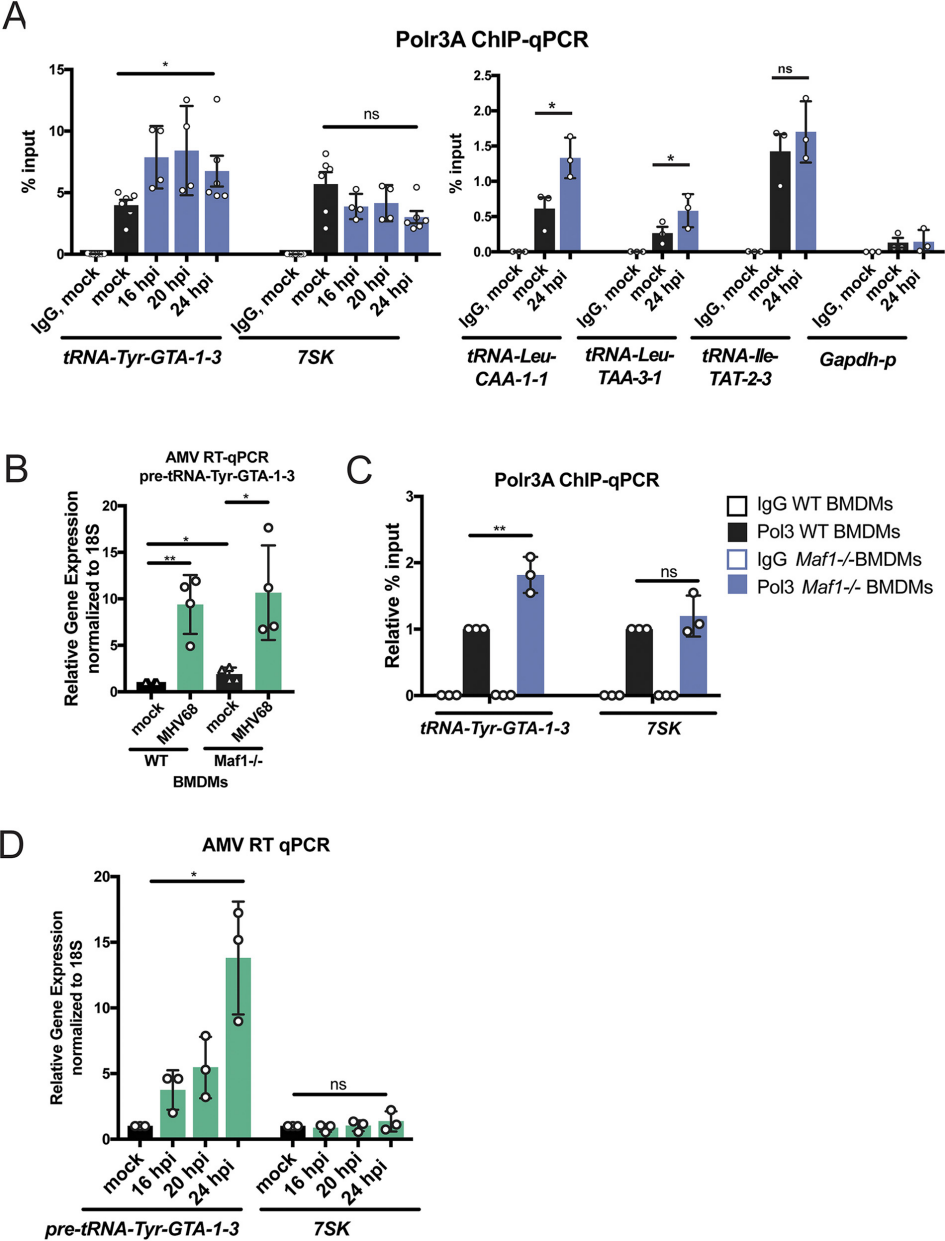
Polr3A is recruited to induced tRNA loci in infected cells. (A) RNAPIII occupancy was measured in mock-infected and MHV68-infected MC57G cells using ChIP-qPCR with antibodies against the Polr3A subunit at three tRNA genes upregulated at the pre-tRNA level by DM-tRNA-seq (tRNA-Tyr-GTA-1-3, tRNA-Leu-CAA-1-1, and tRNA-Leu-TAA-3-1), a tRNA gene not associated with increased pre-tRNA levels (tRNA-Ile-TAT-2-3), *7SK*, and the promoter of RNAPII-driven *Gapdh*. (B) RT-qPCR was performed with RNA extracted from wild-type and *Maf1^−/−^* MHV68-infected bone marrow-derived macrophages (BMDMs). (C) RNAPIII occupancy was measured using Polr3A ChIP-qPCR on wild-type and *Maf1^−/−^* BMDMs at tRNA-GTA-1-3, a tRNA gene also upregulated during MHV68 infection, and *7SK*. Results are reported as relative percentage of input. (D) RNA from mock- or MHV68-infected MC57G cells was extracted at the indicated hours postinfection (hpi) and subjected to RT-qPCR. All infections were done in MC57G fibroblasts at an MOI of 5 for 24 h, except where indicated. Error bars show SD, and statistics were calculated using an unpaired *t* test on Δ*C_T_* values (RT-qPCR) or paired *t* test on raw percentage of input values (ChIP-qPCR). ns, not significant. *, *P* < 0.05, **, *P* < 0.01.

The distinct subset of tRNA genes that have higher expression at the pre-tRNA level during MHV68 infection could be reflective of increased transcription, stability, or a combination of both. To determine if the increase in pre-tRNA transcripts during infection results from transcriptional upregulation, we performed chromatin immunoprecipitation followed by quantitative PCR (ChIP-qPCR) to measure occupancy of the RNAPIII subunit Polr3A at several loci in mock- and MHV68-infected MC57G fibroblasts. RNAPIII abundance specifically increased during MHV68 infection at each of the tested differentially expressed loci (tRNA-Tyr-GTA-1-3, tRNA-Leu-CAA-1-1, and tRNA-Leu-TAA-3-1) but remained unchanged at a tRNA gene not differentially expressed (tRNA-Ile-TAT-2-3) and at *7SK* (Fig. 3A). RNAPIII abundance at the RNAPII-dependent GAPDH (glyceraldehyde-3-phosphate dehydrogenase) promoter was included as a negative control to show specificity of the RNAPIII ChIP signal. These data suggest that there is an increase in RNAPIII recruitment to select tRNA genes during MHV68 infection.

Maf1 is a well-studied negative regulator of transcription by RNAPIII whose activity is controlled by phosphorylation and cellular localization in response to nutrients and stress conditions (41, 42). When active, Maf1 directly targets the Brf1 subunit of TFIIIB and RNAPIII to inhibit transcription. In addition, in proliferating and differentiated mammalian cells, Maf1 chronically represses RNAPIII transcription to balance RNA synthesis with cellular demand (43, 44). Thus, if the mechanism underlying MHV68 induction of pre-tRNAs involved antagonizing Maf1-dependent repression, we reasoned that cells lacking Maf1 should no longer display a difference in pre-tRNA levels between uninfected and infected cells. To test this hypothesis, we derived primary bone marrow-derived macrophages (BMDMs) from WT and *Maf1^−/−^* mice and infected them with MHV68 at a multiplicity of infection (MOI) of 5. MHV68 infection of BMDMs resulted in an ∼10-fold increase in pre-tRNA-Tyr upon MHV68 infection, demonstrating that increased pre-tRNA expression during infection occurs in primary cells and is not unique to fibroblast cell lines (Fig. 3B). MHV68-infected WT and *Maf1^−/−^* BMDMs showed similar levels of pre-tRNA expression, indicating that viral induction of pre-tRNA-Tyr levels was not dependent on *Maf1* regulation of RNAPIII. Similar to other published reports (36), uninfected *Maf1^−/−^* cells showed an ∼2-fold increase in pre-tRNA-Tyr compared to WT BMDMs, as well as increased RNAPIII promoter occupancy in ChIP assays, confirming that RNAPIII activity is elevated in the absence of this negative regulator (Fig. 3B and C).

Even though the relative increases in pre-tRNA-Tyr levels were dramatically larger in MHV68-infected cells compared to cells lacking Maf1 (Fig. 3B and D), we were struck by the observation that the increases in RNAPIII occupancy in these two scenarios were comparable (Fig. 3A and C). To explore the contribution of RNAPIII recruitment to pre-tRNA levels, we compared pre-tRNA abundance by RT-qPCR (Fig. 3D) and RNAPIII promoter occupancy by Polr3A ChIP-qPCR (Fig. 3A) across a time course of MHV68 infection in MC57G fibroblasts. Pre-tRNA-Tyr levels were induced about 5-fold at 16 and 20 h postinfection (hpi) but increased to a nearly 15-fold induction at 24 hpi, whereas *7SK* levels remained constant (Fig. 3D). In contrast to the RNA abundance data, RNAPIII occupancy at the pre-tRNA-Tyr promoter was modestly increased by 16 hpi, but did not further increase at later time points (Fig. 3A). Thus, after 16 hpi, it is likely that posttranscriptional mechanisms underlie the continued accumulation of pre-tRNA-Tyr.

### Virus-induced mRNA degradation contributes to posttranscriptional accumulation of pre-tRNAs.

**FIG 4:**
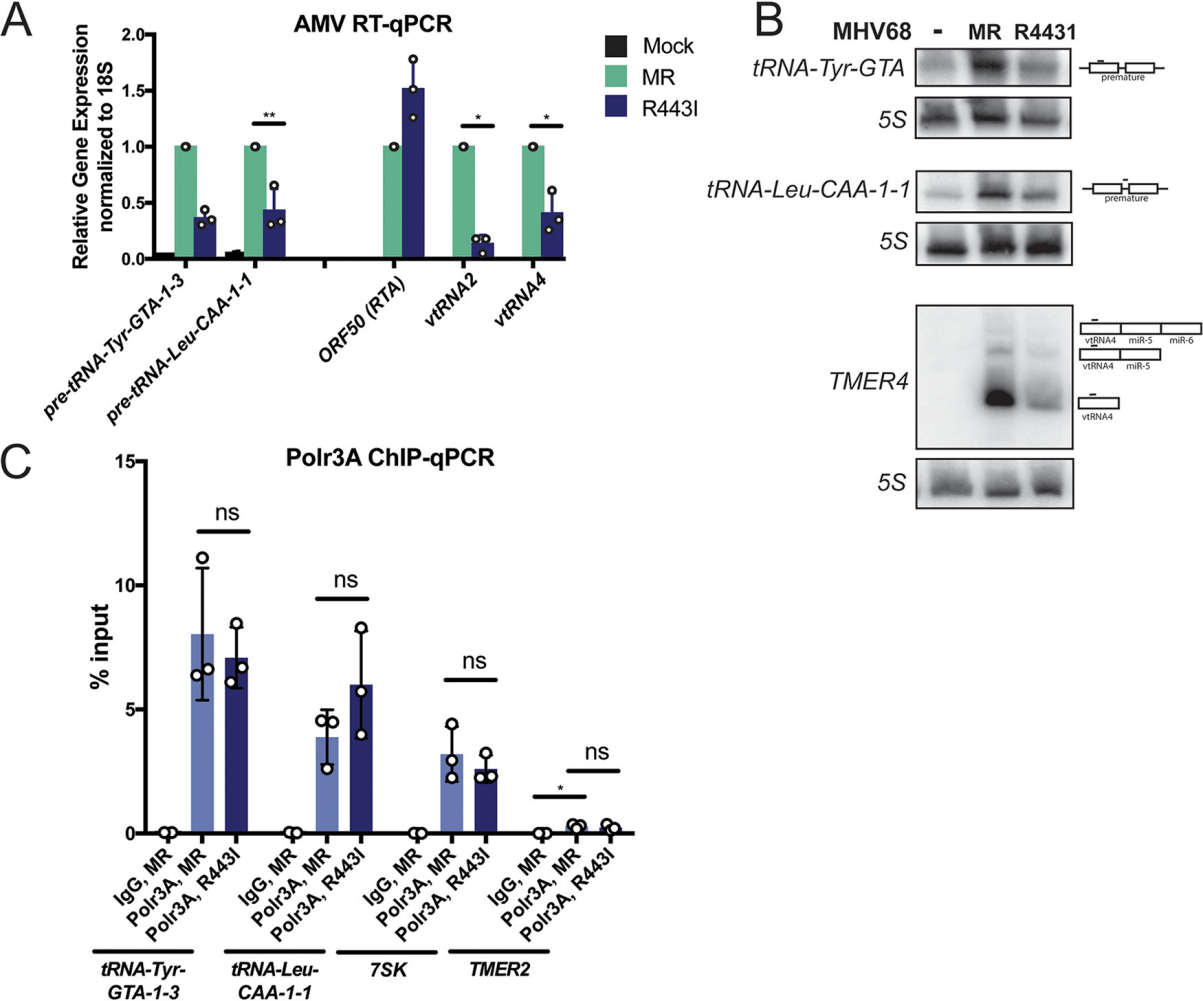
muSOX activity is required for pre-tRNA accumulation, but not for RNAPIII recruitment. (A) MC57Gs were infected with muSOX R443I or mutant revertant (MR) MHV68, and RT-qPCR was performed with extracted RNA. (B) Northern blots were performed on RNA from the same conditions as in panel A using probes to detect pre-tRNA-Tyr, pre-tRNA-Leu-CAA-1-1, and viral TMER4 transcripts. The TMER4 probe binds the vtRNA4 sequence. (C) RNAPIII occupancy was measured using ChIP-qPCR with antibodies against the Polr3A subunit of RNAPIII at induced tRNA loci, viral TMER2, and *7SK* using the cells described in panel A. All infections were done at an MOI of 5 for 24 h. Error bars show SD, and statistics were calculated using an unpaired *t* test on raw Δ*C_T_* values (RT-qPCR) or paired *t* test on raw percentage of input values (ChIP-qPCR). ns, not significant. *, *P* < 0.05, **, *P* < 0.01.

We reasoned that selective posttranscriptional accumulation of pre-tRNAs might occur if pre-tRNA maturation or turnover were impaired: for example, if the virus downregulated factors involved in these processes. A well-characterized phenotype of MHV68 infection is the widespread degradation of host mRNAs, which is caused by the viral mRNA-specific endonuclease muSOX (28). Notably, the levels of the RNAPIII-transcribed viral tRNAs (vtRNAs) are decreased in cells infected with the MHV68 muSOX point mutant R443I (MHV68.R443I), which has reduced host shutoff activity but no viral replication defect in murine fibroblasts (45, 46). muSOX is expressed with early kinetics, but its mRNA depletion phenotype is most prominent at late stages of infection, coincident with maximal pre-tRNA accumulation. We tested whether virus-induced mRNA decay contributes to posttranscriptional accumulation of cellular pre-tRNAs by measuring pre-tRNA abundance in MC57G cells infected with MHV68.R443I or the mutant revertant (MR) virus in which the R443I mutation was restored to wild type (Fig. 4A). Similar to the vtRNAs, the levels of cellular pre-tRNA-Tyr and pre-tRNA-Leu were lower in cells infected with the R443I mutant compared to MR MHV68. In contrast, the viral ORF50 mRNA was slightly increased during the R443I mutant infection, consistent with previous reports (45). We verified these results using Northern blot analysis (Fig. 4B). To determine if these changes reflected transcriptional output, we measured RNAPIII binding at two differentially regulated host tRNA genes and the viral TMER2 locus during MR versus R443I infection (Fig. 4C). RNAPIII abundance was similar during infection with MR and R443I viruses, in stark contrast to the difference in levels of pre-tRNA and vtRNA transcript abundance. This result suggests that the decreased abundance of these transcripts in total RNA is not due to decreased levels of transcription. Collectively, these data reveal that the mRNA decay phenotype associated with WT MHV68 infection is not involved in transcriptional activation of tRNA loci, but instead stabilizes the unprocessed pre-tRNAs.

## DISCUSSION

**FIG 5:**
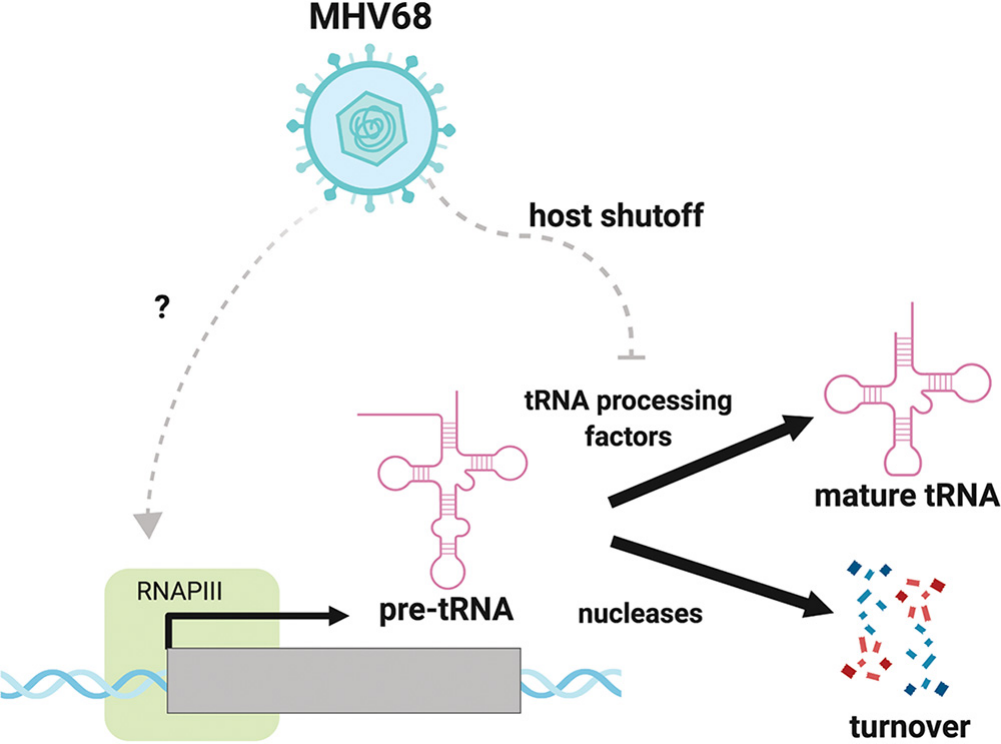
MHV68 infection induces pre-tRNA accumulation by transcriptional and posttranscriptional mechanisms. RNAPIII is recruited to specific tRNA genes by a Maf1-independent mechanism, increasing the output of pre-tRNAs. Pre-tRNAs accumulate in infected cells due to depletion of maturation or turnover machinery during muSOX-mediated mRNA decay (host shutoff).

This study represents the first genome-wide analysis of how infection modulates the tRNAome, revealing that the model gammaherpesvirus MHV68 causes extensive changes to pre-tRNA but not mature tRNA abundance. Additionally, we found evidence that the presence of elevated levels of pre-tRNA-Tyr and -Arg leads to an increase in production of their cognate tRFs. We did not detect increased expression across all tRNA genes, but instead found a dramatic accumulation of pre-tRNAs from a subset of loci, which may reflect differences in promoter accessibility, regulation, and/or pre-tRNA processing. Collectively, our data support a model whereby pre-tRNA accumulation during MHV68 infection is driven by both RNAPIII recruitment and pre-tRNA stabilization due to a block in downstream processing or turnover events (Fig. 5). We hypothesize that early events in infection stimulate RNAPIII-driven transcription of specific tRNA loci, while muSOX-induced depletion or relocalization of pre-tRNA processing and/or turnover machinery stalls further canonical processing or degradation of these transcripts, leading to their buildup late in infection. These findings are bolstered by early reports showing enhanced RNAPIII activity during DNA virus infection, including for select tRNAs (12–17, 27), suggesting that manipulation of the pre-tRNA landscape is likely to be a conserved feature of numerous viruses.

Pre-tRNA steady-state levels are dependent on both the production and stability of the pre-tRNA transcripts. We performed Polr3A ChIP-qPCR and found that increased pre-tRNA abundance was associated with higher levels of Polr3A occupancy during infection. In contrast, a tRNA gene with constant pre-tRNA expression did not show changes in Polr3A binding. This confirms that transcriptional increases—at least at some tRNA genes—contribute to pre-tRNA accumulation during infection. Whether this is true for all tRNA genes associated with increased pre-tRNA levels remains to be determined. Additionally, increased RNAPIII occupancy during infection suggests a mechanism involving RNAPIII recruitment to specific tRNA genes and raises the possibility that these tRNA genes are not expressed to their full potential in the uninfected cell populations. In support of this, there is considerable overlap between tRNA genes upregulated during MHV68 infection with those upregulated in cells lacking Maf1, a negative regulator of RNAPIII that prevents polymerase recruitment. (Fifty-nine of 79 pre-tRNAs induced by MHV68 infection of MC57G fibroblasts were also increased in *Maf1^−/−^* versus WT liver in reference 36.) However, Maf1 does not appear to play a pivotal role in the stark pre-tRNA accumulation seen during the late stages of MHV68 infection (16 to 24 hpi), as the increase in pre-tRNA abundance seen at this time point does not require Maf1.

The mechanisms employed during MHV68 infection to increase RNAPIII recruitment have not been identified. During EBV infection, TFIIIC subunit and Bdp1 protein levels increase, along with transcripts for RNAPIII genes with type I and II promoters (*7SL*, *5S*, and tRNAs), but not those with type III promoters (*7SK*, *U6*, and *MRP*) (12). In contrast, MHV68 infection leads to transcriptional induction of B1/B2 SINEs (20, 34) and certain tRNA genes, all of which have type II promoters. TFIIIB/C subunit mRNA levels do not increase during MHV68 infection, and quantitative mass spectrometry of MHV68-infected cells indicates that total TFIIIB/C protein levels are not elevated, although there may be enrichment of these factors in the nucleus (47). Thus, RNAPIII regulation during MHV68 infection appears distinct from that observed with EBV and may therefore involve novel mechanisms. Like EBV, SV40 infection leads to increased TFIIIC protein levels, but additionally results in higher availability of TFIIIB by large T antigen sequestration of the retinoblastoma tumor suppressor protein (RB), a negative regulator of RNAPIII transcription (48). The changes in RNAPIII output in mouse fibroblasts are similar between SV40 and MHV68 infections (i.e., B1/B2 SINEs and certain tRNAs are induced [27, 49]); additionally, mass spectrometry data suggest that the RB subcellular concentration changes during MHV68 infection (47). Thus, future studies will explore how RB is regulated during MHV68 infection and whether this protein is an important player in gammaherpesvirus-induced RNAPIII activation. Potential roles of RB could include the posttranslational regulation of transcription factor activities, altered factor availability, or epigenetic changes influencing gene accessibility.

With the exception of selenocysteine tRNA (7), all tRNA genes use the same internal A/B box promoter architecture for transcription. However, based on differential binding of RNAPIII, many studies have revealed that tRNA genes are not equally transcribed in mammalian cells, as measured by the differential binding of RNAPIII (7–9). Genome-wide studies examining chromatin accessibility, modification, and transcription factor binding have suggested that a driver of RNAPIII occupancy at tRNA genes is open and active chromatin, typically in the vicinity of RNAPII transcription sites, yet the basis for differential RNAPIII binding remains to be determined (7, 8, 50, 51). Further studies are needed to assess how host chromatin accessibility and modifications change during MHV68 infection. Interestingly, MHV68 infection leads to depletion of RNAPII-specific subunits, resulting in less RNAPII transcription of the host genome during infection (47). There is some evidence of transcriptional interference between RNAPIII and RNAPII bound at nearby genes, as depletion of the Rbp1 subunit of RNAPII leads to upregulation of a subset of tRNA genes (52). It is possible that depletion of RNAPII allows for increased recruitment of RNAPIII machinery. We hypothesize that a combination of genome accessibility and transcription factor availability are associated with tRNA transcriptional increases during infection.

Although tRNA gene transcription is elevated due to RNAPIII recruitment, the magnitude of pre-tRNA abundance is unlikely to be explained by transcriptional activation alone. Instead, our data suggest that pre-tRNA stability is also increased. While we have not been able to measure pre-tRNA half-life directly (due to difficulty in robustly inhibiting RNAPIII in the required short time frame of pre-tRNA processing), it is clear that pre-tRNAs accumulate dramatically between 20 and 24 hpi, a time frame in which RNAPIII occupancy is unchanged. Infection-induced pre-tRNAs do not seem to be processed into mature tRNAs, as we found no evidence of differential expression at the mature tRNA transcript level. Similarly, *Maf1^−/−^* tissues exhibit upregulation of pre-tRNAs without significant changes to mature tRNA levels, suggestive of a homeostatic mechanism to maintain the size of mature tRNA pools when tRNA gene transcription is increased (36). In both of these cases, it is likely that the increase of pre-tRNAs upon transcriptional induction saturates processing machinery, an outcome well established in yeast (53, 54). It was striking that some pre-tRNA transcripts revealed in our study were much more highly expressed than others. These pre-tRNAs might have specific modifications, unique structural characteristics, or processing fates that render them inherently more stable in the cell or during infection. Additionally, excess pre-tRNAs might be cleaved into stable, and perhaps functionally distinct, fragments. We demonstrated here one example of a tRF derived from pre-tRNA-Tyr that is elevated during MHV68 infection. This Tyr tRF was originally described to be abundant in the absence of the Clp1 kinase, a known regulator of the TSEN tRNA splicing complex (38, 55, 56). As the 3′ end of this tRF corresponds to the exact end of the 5′ exon boundary (38), it is possible that the Tyr and Arg tRFs are splicing intermediates and thus constitute evidence that splicing of pre-tRNA transcripts is indeed perturbed upon infection. However, as only a small fraction of pre-tRNAs undergo splicing, there are likely many aspects of pre-tRNA maturation that are perturbed in the infection context. Regardless, knowing the identity of hyperabundant pre-tRNAs will help guide studies to investigate unique features and broaden our understanding of tRNA maturation and turnover.

We hypothesized that tRNA processing and turnover machinery might be depleted in infected cells due to gammaherpesvirus-induced host shutoff implemented at late stages of viral infection. Widespread mRNA decay is initiated by a gammaherpesvirus-encoded endonuclease, called SOX in KSHV and muSOX in MHV68. SOX and its homologs cleave RNAPII-transcribed mRNAs, leading to subsequent downregulation of host protein synthesis (28, 57). Depletion of host proteins might further increase the ratio of pre-tRNA to available processing and turnover machinery, leading to increased pre-tRNA half-lives. Though total protein levels of tRNA processing machinery are not dramatically altered during a 24-h lytic infection cycle, there are some decreases in nuclear abundance of tRNA-related factors during infection, including the RNA exosome (47), which participates in pre-tRNA surveillance and turnover in murine cells (58). Modest changes in abundance of many tRNA-related processing and turnover complexes could have an additive effect on the stability of pre-tRNAs in infected cells. Moreover, pre-tRNAs were less abundant in cells infected with the host shutoff-defective R443I virus, a phenotype not explained by changes in Polr3A abundance at tRNA genes. Viral TMER transcripts are also less abundant under these conditions (45), suggesting that the factors involved with pre-tRNA stability during infection may also be involved in viral TMER transcript stability. TMERs consist of a tRNA-like sequence with 1 to 2 microRNA (miRNA) stem loops that are processed by the host tRNA 3′ processing enzyme tRNAse Z (59, 60). Whether tRNAse Z or other host factors are involved in stabilizing host pre-tRNA and viral TMERs is an important question for future studies.

Boosting RNAPIII activity is thought to promote the expression of RNAPIII-transcribed genes carried by some DNA viruses (e.g., Epstein-Barr virus-encoded small RNAs [EBERs], adenovirus virus-associated [VA] RNAs, and MHV68 TMERs); however, not all DNA viruses carry RNAPIII genes, suggesting that regulation of RNAPIII could be advantageous to the virus or host for other reasons. For example, because nascent RNAPIII transcripts contain 5′ triphosphate ends, they can and do serve as the substrates for RNA sensors in the cell, such as RIG-I (18, 19). These studies show that while typically the 5′ ends of RNAPIII transcripts are processed or protected from sensing by protein binding partners, HSV-1 and KSHV infection tips the balance and results in exposure of 5′ triphosphate ends of RNAPIII transcripts for RIG-I recognition. Additionally, increased tRNA fragmentation observed during MHV68 infection could result in modulation of gene regulation, cell proliferation, and stress responses, as has been reported in for tRFs produced during viral infection or cancer (5, 6). A thorough examination of tRFs produced during gammaherpesvirus infection by small RNA-seq will be required to begin to address the role of tRF generation upon infection. In summary, exploring viral modulation of host tRNAomes could lead to exciting insights both for tRNA biology and for the role of RNAPIII activation during DNA virus infection.

## MATERIALS AND METHODS

### Cell and virus culture.

NIH 3T3 and MC57G cell lines (ATCC) were maintained in Dulbecco's modified Eagle medium (DMEM; Gibco) plus 10% fetal bovine serum (FBS; VWR) and screened regularly for mycoplasma by PCR. Wild-type and *Maf1* knockout primary bone marrow-derived macrophages (BMDMs) were differentiated from adult 3- to 6-month old C57BL/6 mice (36). Provided femurs and tibias were ground and passed through a 70-μm-pore filter to remove debris. Cells were differentiated in macrophage medium (high-glucose DMEM, 10% FBS, 10% macrophage colony-stimulating factor [M-CSF], 1% GlutaMax) plus 1% penicillin-streptomycin (PenStrep) for 7 days. MHV68 was amplified in NIH 3T12 cells, and the 50% tissue culture infective dose (TCID_50_) was measured on NIH 3T3 cells by limiting dilution. Green fluorescent protein (GFP)-expressing wild-type (61) and GFP-expressing ΔHS (SOX R443I [46]) MHV68 viruses were incubated with cells for 1 h at an MOI of 5 to allow viral entry, and then the virus-containing medium was removed and replaced with fresh medium. PAA was used at a concentration of 200 μg/ml and was added at the start of the infection. UV inactivation of MHV68 was performed by autocross-linking twice in a Stratalinker 2400 (1,200 μJ × 100). For infection of BMDMs, virus was added to cells in serum-free DMEM for 4 h in non-treated cell culture plates. Virus-containing medium was then aspirated and replaced with macrophage medium without antibiotics.

### RNA isolation and analysis.

Total RNA was isolated from cells using TRIzol (Invitrogen), treated with Turbo DNase (Ambion), and reverse transcribed with AMV RT (Promega) primed with random 9-mers. For TGIRT-qPCR, cDNA was synthesized with the TGIRT-III enzyme (InGex) following the manufacturer’s instructions. Quantitative PCR analysis was performed with iTaq Universal SYBR green Supermix (Bio-Rad) using the primers listed in Table S1 in the supplemental material. qPCR was performed on at least three biological replicates and threshold cycle (*C_T_*) values were measured from three technical replicates per biological sample. Fold change was calculated by ΔΔ*C_T_* method. For Northern blots, 20 μg of RNA was loaded into 8 to 12% PAGE–7 M urea gels and transferred to Hybond-XL or N+ (GE) membranes using a Trans-Blot Turbo Transfer system (Bio-Rad). Blots were prehybridized in ULTRAhyb buffer (Thermo Fisher) at 42°C for 1 h before adding radiolabeled probe. Probes were generated by end labeling oligonucleotides listed in Table S1 using T4 PNK and [γ-^32^P]ATP. Blots were probed overnight at 42°C and washed 3 times for 5 min in 0.5× SSC (1× SSC is 0.15 M NaCl plus 0.015 M sodium citrate) and 0.1% SDS. Primer extension was performed as described in reference 62.

10.1128/mBio.02664-20.5 TABLE S1 Oligonucleotides used in this study. Download Table S1, DOCX file, 0.01 MB. Copyright © 2020 Tucker et al. 2020 Tucker et al. This content is distributed under the terms of the Creative Commons Attribution 4.0 International license.

### DM-tRNA-seq library preparation.

The protocol for library preparation was modified from reference 3. His-tagged wild-type and D135S AlkB plasmids were obtained from Addgene (no. 79050 and 79051), and proteins were purified by a Ni-nitrilotriacetic acid (NTA) column followed by cation exchange. Total RNA extracted from four biological replicates was spiked with *in vitro*-transcribed (IVT) Escherichia coli tRNA-Lys, E. coli tRNA-Tyr, and Saccharomyces cerevisiae tRNA-Phe transcripts at 0.01 pmol IVT tRNA per μg total RNA. RNA was deacylated in 0.1 M Tris-HCl (pH 9) at 37°C for 45 min, dephosphorylated with PNK, and then purified with a mirVANA small RNA purification kit (Ambion) to isolate RNAs of ≤200 nt. Small RNAs were demethylated in a mixture of 300 mM NaCl, 50 mM morpholineethanesulfonic acid (MES; pH 5), 2 mM MgCl_2_, 50 μM ferrous ammonium sulfate, 300 μM 2-ketoglutarate, 2 mM l-ascorbic acid, 50 μg/ml bovine serum albumin (BSA), 1 U/μl SUPERasin, a 2× molar ratio of WT AlkB, and a 4× molar ratio of D135S AlkB for 2 h at room temperature. The reaction was quenched with 5 mM EDTA and purified with TRIzol. A total of 100 ng demethylated small RNAs was used for library preparation with a TGIRT Improved Modular Template-Switching RNA-seq kit (InGex) following the manufacturer’s instructions. The included RNA/DNA heteroduplex for the template switching reaction by TGIRT contains a 3′ N-overhang on the DNA primer to promote reverse transcription of all small RNAs (as opposed to the T-overhang for targeting mature tRNAs described in reference 3). PCR amplification was performed with Phusion polymerase (Thermo Fisher) with Illumina multiplex and barcoded primers synthesized by IDT for 12 cycles. The library was sequenced on a HiSeq4000.

### DM-tRNA-seq bioinformatic analysis.

tRNAscan-SE (63) was used to create the predicted tRNA gene library. Pre-tRNA sequences were defined by adding 50 bp of genomic sequences on 5′ and 3′ ends of the predicted tRNA genes. Mature tRNAs were appended with a CCA tail and were clustered as described in reference 64. Illumina 150-bp paired-end sequence data were subjected to quality control using BBDuk (https://jgi.doe.gov/data-and-tools/bbtools/). Sequencing adapters were removed from raw reads. Bases that have a quality score lower than 30 were trimmed. Any reads that are at least 50 bp in length after quality control were aligned to the mature tRNA reference using the end-to-end mapping mode in Bowtie2 (65) (version 2.3.4.1). Reads that did not map to mature tRNAs were then mapped to the masked mm10 genome appended with pre-tRNA sequences. Two approaches were used for this mapping step. One was to use Bowtie2, with reads that map to multiple locations excluded from downstream analysis, followed by quantification of expression using SALMON (33). The other approach used SALMON to map the reads and quantify the expression levels, which includes the reads that map to multiple locations in estimating the expression levels. Raw counts were normalized with spike-in RNA species, using the R package RUVSeq (66). A generalized linear model was built to test for differential expression for each RNA species, using the R package edgeR (67, 68).

### Chromatin immunoprecipitation.

Cells from a 10-cm dish were cross-linked in 1% formaldehyde in phosphate-buffered saline (PBS) for 5 min at room temperature, quenched in 0.125 M glycine, and washed twice with PBS. Cross-linked cells were lysed with 1 ml ChIP lysis buffer (50 mM HEPES [pH 7.9], 140 mM NaCl, 1 mM EDTA, 10% glycerol, 0.5% NP-40, 0.25% Triton X-100) by rotation for 10 min. Nuclei were collected by centrifugation at 1,700 × *g* for 5 min at 4°C, washed once with ChIP shearing buffer (50 mM Tris Cl, pH 7.5, 10 mM EDTA, 0.1% SDS), and then resuspended in 1 ml of ChIP shearing buffer. Chromatin was sheared for 8 min using a Covaris S220 focused ultrasonicator at 140 power, a 5% duty cycle, and 200 bursts/cycle. Chromatin was spun at 15,000 × *g* for 5 min at 4°C, and the pellet was discarded. Forty micrograms of chromatin was incubated with 10 μg rabbit polyclonal anti-POLR3A (Abcam ab96328) or rabbit IgG (Southern Biotech) overnight. Thirty microliters of mixed protein A and G Dynabeads (Thermo Fisher) was added, and the tubes were rotated for 2 h at 4°C. Beads were washed with low-salt immune complex (20 mM Tris [pH 8.0], 1% Triton X-100, 2 mM EDTA, 150 mM NaCl, 0.1% SDS), high-salt immune complex (20 mM Tris [pH 8.0], 1% Triton X-100, 2 mM EDTA, 500 mM NaCl, 0.1% SDS), lithium chloride immune complex (10 mM Tris [pH 8.0], 0.25 M LiCl, 1% NP-40, 1% deoxycholic acid, 1 mM EDTA), and Tris-EDTA for 5 min each at 4°C with rotation. DNA was eluted from the beads using 100 μl of elution buffer (150 mM NaCl, 50 μg/ml proteinase K) and incubated at 50°C for 2 h and then at 65°C overnight. DNA was purified using a Zymo Oligo Clean and Concentrator kit and used for qPCR analysis with the primers listed in Table S1.

### Replicates and statistical analysis.

This work contains cell culture-based assays, where biological replicates are defined as experiments performed on at least three distinct samples (cells maintained in different flasks). Technical replicates are defined as experiments performed on the same biological sample at least three times. The data point for each biological replicate performed is depicted in the figures. Statistical analysis was performed using Prism 7 (Version 7.0c) software (GraphPad Software), and the exact test performed is described in the figure legends. The criteria for including data involved the quality of the data for positive and negative controls.

### Data availability.

Data have been deposited at NCBI GEO under accession no. GSE142393.
