# Supplementary figures and images for: Alteration of the Premature tRNA Landscape by Gammaherpesvirus Infection
Source: mBio. 2020 Dec 15;11(6):e02664-20. doi: 10.1128/mBio.02664-20 (PMC7773990; doi:10.1128/mBio.02664-20)

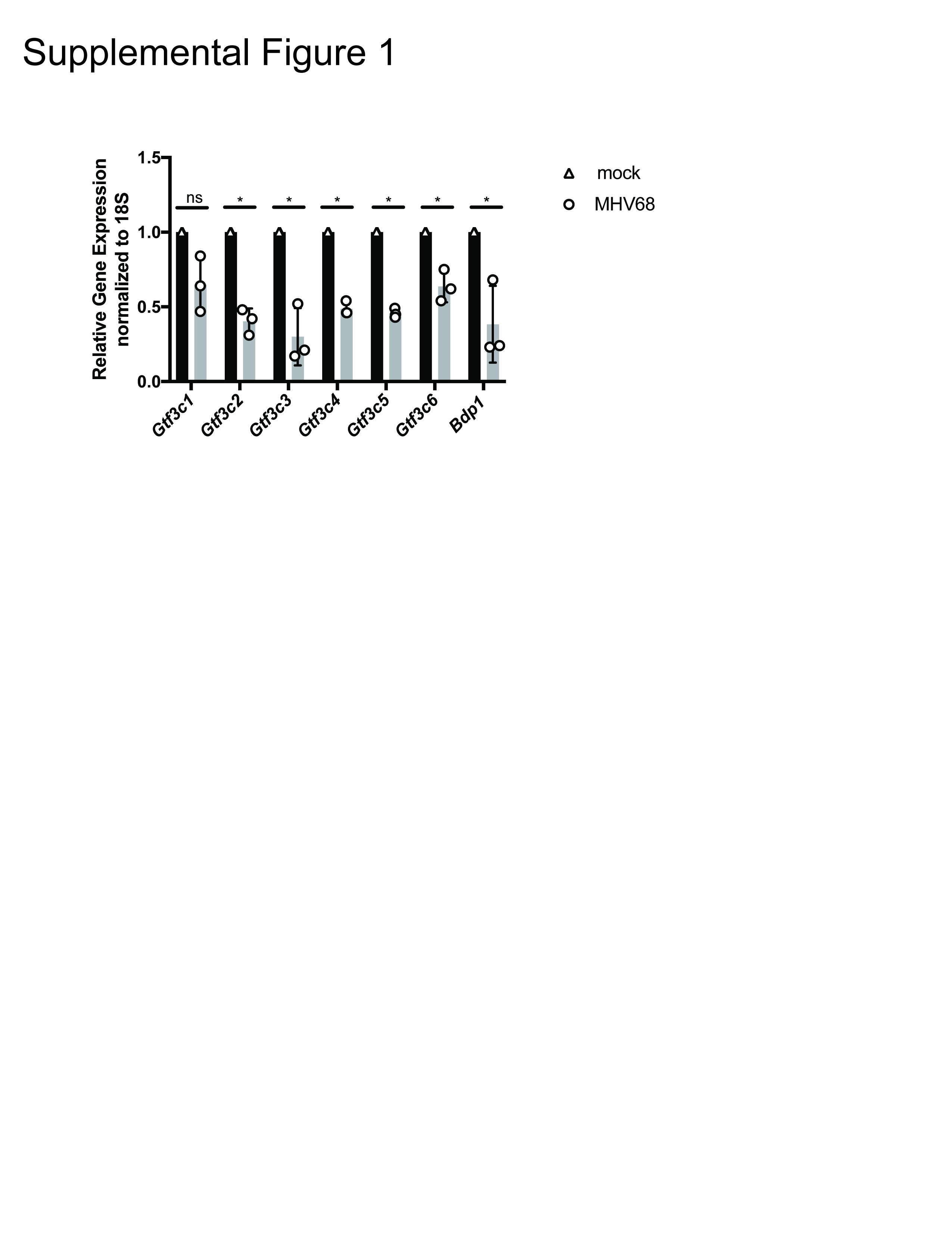

Supplement: FIG S1 [file mBio.02664-20-sf001.tif]

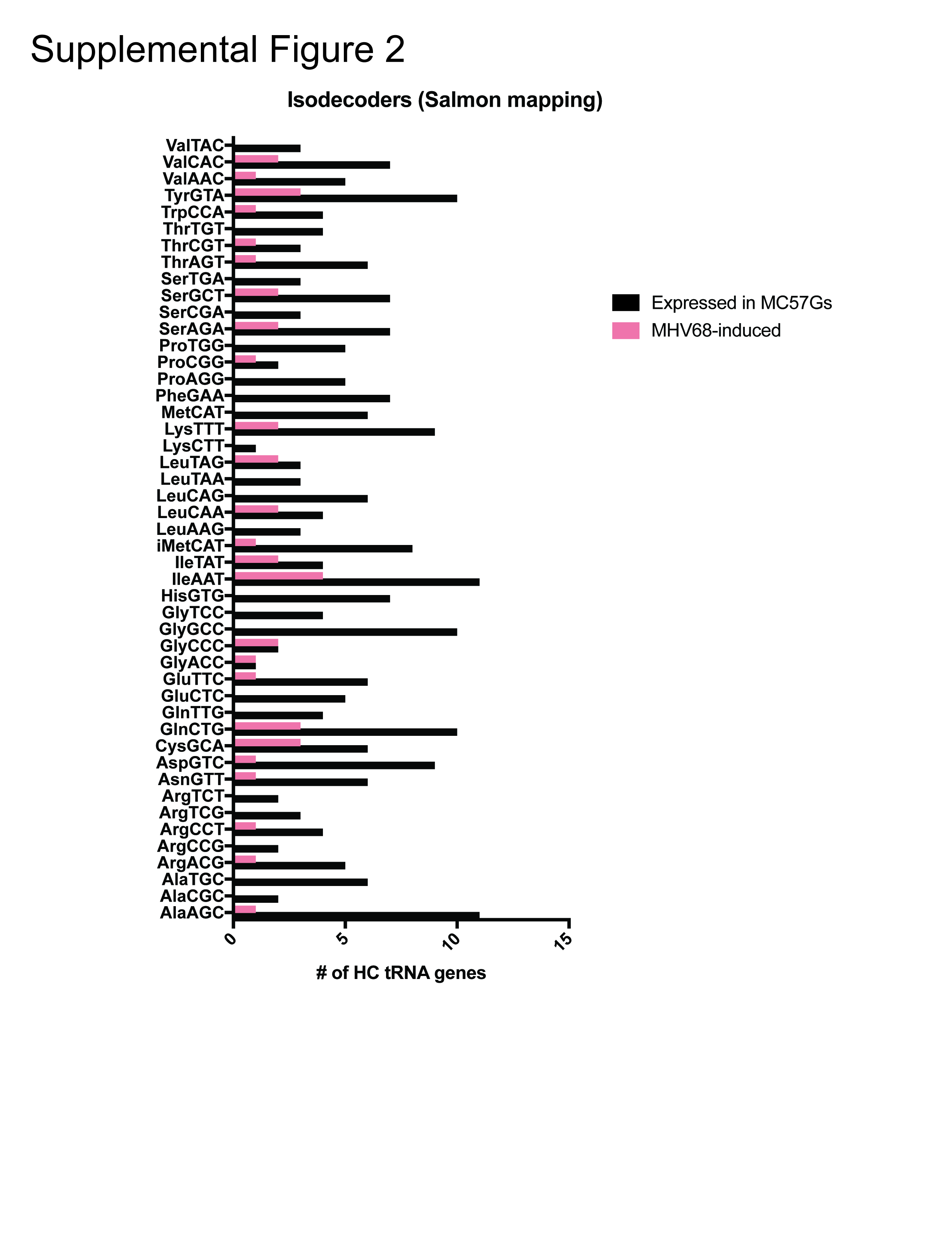

Supplement: FIG S2 [file mBio.02664-20-sf002.tif]

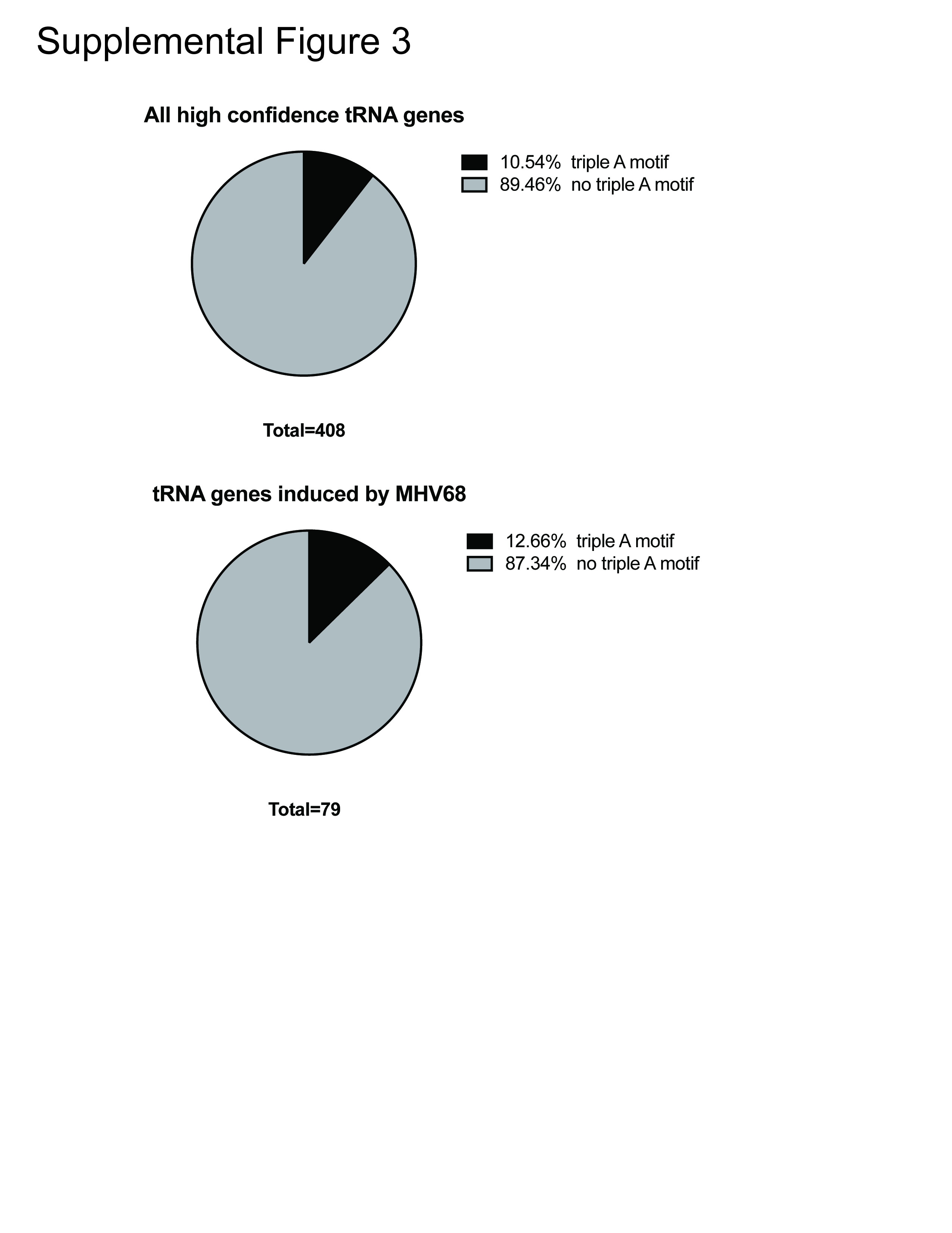

Supplement: FIG S3 [file mBio.02664-20-sf003.tif]

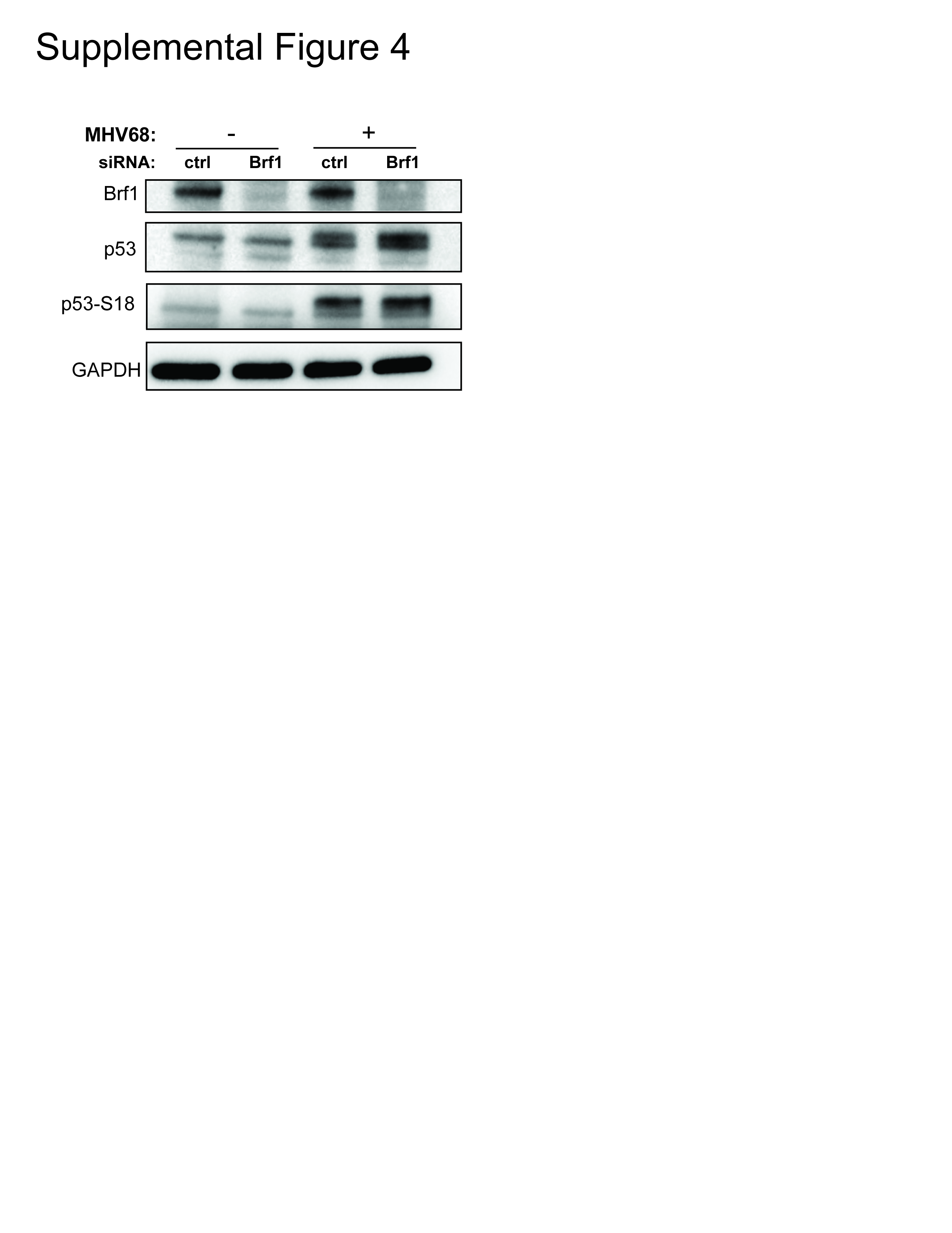

Supplement: FIG S4 [file mBio.02664-20-sf004.tif]
