# Supplementary material for: Alteration of the Premature tRNA Landscape by Gammaherpesvirus Infection
Source: mBio. 2020 Dec 15;11(6):e02664-20. doi: 10.1128/mBio.02664-20 (PMC7773990; doi:10.1128/mBio.02664-20)
Supplement: TABLE S1 [file mBio.02664-20-st001.docx]

**Supplemental Table 1. Oligos used in this study.**

| **Primer extension oligos (mouse)** |  |
| --- | --- |
| B2_PXT | TACACTGTAGCTGTCTTCAGACA |
| 7SK_PXT | GAGCTTGTTTGGAGGTTCT |
| **Northern probes** |  |
| tRNA-Leu-CAA-1-1_intron | CCCTCAGAGCGAGGAAGCCATAG |
| tRNA-Tyr-GTA_5’exon | CTACAGTCCTCCGCTCTACCA |
| tRNA-Leu-TAA-3-1_3’trailer | GCAGACAGTCGAGCAGTACA |
| tRNA-Leu-TAA-3-1_gene | CAGGCAACCGCCCATTGGAT |
| tRNA-Phe-GAA | GATCTTCAGTCTAACGCTC |
| tRNA-Cys-GCA | GaCCTCTTGATCTGCAGTCA |
| tRNA-Lys-TTT | CAGGGACTTGAACCCTGGaC |
| tRNA-Arg | TAGAAGTCCAATGCGCTATCC |
| TMER4/vtRNA4 | ATCTCAACTCTGCGTCGG |
| 5S | AGCCTACAGCACCCGGTATT |
| **RT-qPCR primers (mouse)** |  |
| Mm_pre-tRNA-Tyr-GTA-1-3_F | CCTTCGATAGCTCAGTTGGTAGAGC |
| Mm_pre-tRNA-Tyr-GTA-1-3_R | CTTCGAGCCGGATTCGAACCAGCGAC |
| Mm_pre-tRNA-Leu-CAA-1-1_F | CGCCAGACTCAAGCTATCGC |
| Mm_pre-tRNA-Leu-CAA-1-1_R | TGTCAGAAGTGGGATTCGAACC |
| 7SK_F | CCCCTGCTAGAACCTCCAAAC |
| 7SK_R | CACATGCAGCGCCTCATTT |
| U6_F | CGCTTCGGCAGCACATATAC |
| U6_R | AAAATATGGAACGCTTCACGA |
| 5S_F | TCTCGTCTGATCTCGGAAGC |
| 5S_R | AGCCTACAGCACCCGGTATT |
| 7SL_F | ATCGGGTGTCCGCACTAAGTT |
| 7SL_R | CAGCACGGGAGTTTTGACCT |
| GTF3C1_F | TTCACACACCGCTTTCAAG |
| GTF3C1_R | CTCCAGTAGGTAATCGGATCACA |
| GTF3C2_F | ACAGCAAGAGGCGCTAAAAGG |
| GTF3C2_R | GGACTGATCTAAGAGACCAGGAA |
| GTF3C3_F | ATGGGGTGAGTAAGTCAGTTCCA |
| GTF3C3_R | TCCAACACAAATACATCGCCTG |
| GTF3C4_F | CCCAATGCGGGGTTTCAAATA |
| GTF3C4_R | CTGCACAGTCAGGCGATTGT |
| GTF3C5_F | TTGGTGTGCGTGGAGTACC |
| GTF3C5_R | CCCAGGGTCTGTAGCATCT |
| GTF3C6_F | GCGTCTCCGAACGCTATGG |
| GTF3C6_R | CCTCTCCGTGTCAATTCCCA |
| BDP1_F | TGCTTCTTCTGAGATAGGGCA |
| BDP1_R | GAGGGCTTCTTTAGCAGCACT |
| **RT-qPCR primers (MHV68)** |  |
| MHV68_orf50_F | GGCCGCAGACATTTAATGAC |
| MHV68_orf50_R | GCCTCAACTTCTCTGGATATGCC |
| MHV68_gB_F | GGCCCAAATTCAATTTGCCT |
| MHV68_gB_R | CCCTGGACAACTCCTCAAGC |
| MHV68_vtRNA2_F | GGTAGAGCAGCGGTTCCT |
| MHV68_vtRNA2_R | ACTCCCCCTCTCAACCA |
| MHV68_vtRNA4_F | AGCGGCAGGCTCATC |
| MHV68_vtRNA4_R | ATCTCAACTCTGCGTCGG |
| **ChIP-qPCR primers (mouse)** |  |
| Mm_tRNA-Tyr-GTA-1-3_F | CCTTCGATAGCTCAGTTGGTAGAGC |
| Mm_tRNA-Tyr-GTA-1-3_R | CTTCGAGCCGGATTCGAACCAGCGAC |
| Mm_tRNA-Leu-CAA-1-1_F | CGCCAGACTCAAGCTATCGC |
| Mm_tRNA-Leu-CAA-1-1_R | TGTCAGAAGTGGGATTCGAACC |
| Mm_tRNA-Leu-TAA-3-1_F | ACTGGGATGGCTGAGTGGTTAAGGC |
| Mm_tRNA-Leu-TAA-3-1_R | GCAGACAGTCGAGCAGTACA |
| Mm_tRNA-Ile-TAT-2-3_F | GCTCCAGTGGCGCAATCGGTTAGC |
| Mm_tRNA-Ile-TAT-2-3_R | GCCTAGCTCCAAAAGGATCG |
| 7SK_F | CCCCTGCTAGAACCTCCAAAC |
| 7SK_R | CACATGCAGCGCCTCATTT |
| GAPDH_promoter_F | GGTTATCACCAGGCCAGCTA |
| GAPDH_promoter_R | GGAAGGGAGAAAAGGCATTC |
| **ChIP-qPCR primers (MHV68)** |  |
| MHV68_vtRNA2_F (TMER2) | GGTAGAGCAGCGGTTCCT |
| MHV68_vtRNA2_R (TMER2) | ACTCCCCCTCTCAACCA |
